# Polyphenols from Chilean Propolis and Pinocembrin Reduce MMP-9 Gene Expression and Activity in Activated Macrophages

**DOI:** 10.1155/2016/6505383

**Published:** 2016-03-28

**Authors:** Nicolás Saavedra, Alejandro Cuevas, Marcela F. Cavalcante, Felipe A. Dörr, Kathleen Saavedra, Tomás Zambrano, Dulcineia S. P. Abdalla, Luis A. Salazar

**Affiliations:** ^1^Center of Molecular Biology and Pharmacogenetics, Department of Basic Sciences, Scientific and Technological Bioresource Nucleus, Universidad de La Frontera, Avenida Francisco Salazar 01145, 4811230 Temuco, Chile; ^2^Department of Clinical and Toxicological Analyses, Faculty of Pharmaceutical Sciences, Universidade de São Paulo, Avenida Professor Lineu Prestes 580, 05508-000 São Paulo, SP, Brazil

## Abstract

Polyphenols from diverse sources have shown anti-inflammatory activity. In the context of atherosclerosis, macrophages play important roles including matrix metalloproteinases synthesis involved in degradation of matrix extracellular components affecting the atherosclerotic plaque stability. We prepared a propolis extract and pinocembrin in ethanol solution. Propolis extract was chemically characterized using LC-MS. The effect of treatments on gene expression and proteolytic activity was measured* in vitro* using murine macrophages activated with LPS. Cellular toxicity associated with both treatments and the vehicle was determined using MTT and apoptosis/necrosis detection assays. MMP-9 gene expression and proteolytic activity were measured using qPCR and zymography, respectively. Thirty-two compounds were identified in the propolis extract, including pinocembrin among its major components. Treatment with either ethanolic extract of propolis or pinocembrin inhibits MMP-9 gene expression in a dose-dependent manner. Similarly, an inhibitory effect was observed in proteolytic activity. However, the effect showed by ethanolic extract of propolis was higher than the effect of pinocembrin, suggesting that MMP-9 inhibition results from a joint contribution between the components of the extract. These data suggest a potential role of polyphenols from Chilean propolis in the control of extracellular matrix degradation in atherosclerotic plaques.

## 1. Introduction

Matrix metalloproteinases (MMPs) are a family of proteolytic enzymes involved in physiological processes associated with homeostasis regulation, host defense, and tissue repair. These proteins belong to a family of calcium-dependent, zinc-containing endopeptidases that degrade proteins and proteoglycan components of extracellular matrix (ECM) [1]. Diverse cellular types, including connective tissue cells, proinflammatory cells, osteoblasts, endothelial cells, neutrophils, lymphocytes, and macrophages, express MMPs. Regularly, the expression of these enzymes in physiological mechanisms is under strict control, playing an important role in ECM remodeling under normal conditions such as fetal tissue development and postnatal tissue repair [2]. In pathological events, deregulation of MMPs is frequent [3], and excessive breakdown of ECM is observed in connective tissue destruction and remodeling associated with cancer invasion and metastasis [4], cartilage destruction in arthritis [5], and atherosclerotic plaque rupture [6]. More specifically, the deregulation of MMP-9 expression has been associated with tumor invasiveness [4, 7, 8], atherosclerotic plaque rupture in animals with advanced lesions [9], and acute coronary syndrome in humans [10]. MMP-9 or 92-kDa gelatinase is expressed by activated macrophages and foam cells in atheroma plaque [11] and is specialized in the digestion of basement membrane collagens and elastin, facilitating macrophage extravasation [12, 13]. MMP-9 expression is increased in inflammatory, malignant, and degenerative diseases, particularly in acute coronary syndrome in humans, where circulating MMP-9 levels are increased [10], suggesting that inhibition of MMP-9 activity might have a therapeutic potential.

Propolis is a polyphenol-rich resinous substance collected by honeybees from a variety of plant sources as trees and shrubs. Its colour is variable depending on the plant from which is collected, and its smell is intense and aromatic [14]. It is generally composed by fats, aliphatic and aromatic hydrocarbons, flavonoids, alcohols, terpenes, sugars, and esters. Its chemical composition is very complex and varies according to geographic origin depending on the local flora from which it was produced [15, 16], as well as bee species that performed the collection [17]. This variability results in differences between the biological properties showed by different extracts [18]. Propolis has been used as a complementary medicine since ancient times [19], demonstrating biological activity such as lipid lowering effects and antibacterial, antitumor, and anti-inflammatory effects [20–24]. In our country, there are reports of antifungal activity against* Candida *spp. [25] and hepatoprotective [26] and antioxidant activities [27]. Chemical characterization of Chilean propolis has identified pinocembrin among its main components, which also showed biological activity as an isolated compound [28–30]. Pinocembrin (5,7-dihydroxyflavanone) is one of the primary flavonoids in propolis, can be extracted as a pure compound, and has been incorporated in pharmaceutical industry for its wide range of pharmacological effects [30], including antimicrobial, anti-inflammatory, antioxidant, and anticancer activities [31–33].

Considering these antecedents, the aim of the present study was to evaluate the effect of polyphenols from Chilean propolis and pinocembrin on MMP-9 gene expression and gelatinolytic activity in activated macrophages.

## 2. Materials and Methods

### 2.1. Ethanolic Extract of Propolis (EEP) and Pinocembrin

An ethanolic extract was prepared from a propolis sample obtained from southern Chile (Cunco, La Araucanía). The sample was desiccated and frozen at −20°C. Then, the propolis (150 g) was pounded and macerated in 80% ethanol (v/v) protected from light for 30 minutes at 60°C under constant shaking. The resulting solution (EEP) was centrifuged (5000 ×g for 5 minutes) and filtered using first an AP20 prefilter (Millipore, USA) and then a 0.2 *μ*m hydrophobic filter (Millipore, USA), both in a vacuum filtration system. Later, the solvent was removed using a rotary evaporator. Finally, the resulting material was dissolved in a reduced volume of 80% ethanol (v/v) in order to obtain an EEP in which the solvent does not exceed 0.02% v/v of final concentration in the culture medium. Pinocembrin (Sigma-Aldrich Chemie GmbH, Schnelldorf, Germany) was dissolved using the same solvent as EEP.

### 2.2. EEP Chemical Characterization

The EEP was characterized by liquid chromatography coupled to diode array detection and mass spectrometry (LC-DAD-MS). Chromatographic separation was achieved in a Shimadzu Prominence (Kyoto, Japan) using a C-18 column (250 × 3.0 mm, 5 *μ*m; Luna C-18(2), Phenomenex, CA, USA) at 40°C. Gradient elution employed (A) water and (B) methanol both with 0.1% formic acid (30 to 60% B in 80 min, 0.5 mL/min). Mass spectrometry data was acquired with an Esquire HCT (Bruker Daltonics, MA, USA) after electrospray ionization in positive and negative modes. UV absorption profiles and fragmentation results (MS/MS) were compared to literature for compound assignments.

### 2.3. Cell Culture

RAW 264.7 cells were maintained in RPMI 1640 supplemented with 10% heat-inactivated fetal bovine serum (FBS), 2.0 g/L sodium bicarbonate, 16.5 mmol/L HEPES, and 1x antibiotic/antimicotic mixture at 37°C in a 5% CO_2_ humidified atmosphere.

### 2.4. Cell Viability

To evaluate the effect of treatments on cell viability, we used the 3-(4,5-dimethylthiazol-2-yl)-2,5-diphenyl tetrazolium bromide (MTT) reduction assay. RAW 264.5 cells (5.0 × 10^3^/well) were seeded in a 96-well plate in the conditions above described, 12 hours before the experiment. The cells were treated with EEP or pinocembrin at concentrations within 1–10 *μ*g/mL in presence or absence of LPS stimuli (100 ng/mL) during 12 hours. The effect of vehicle was also tested using it like treatments. Then, the medium was replaced by RPMI-without phenol red containing MTT (1 mg/mL, Sigma Chemical Company, St. Louis, MO, USA) and the cells were incubated for 3 hours. After supernatant discarding, the precipitate was dissolved with dimethyl sulfoxide and the optical density was measured at 580 nm using a spectrophotometric microplate reader. Relative viability percentage with respect to the control cells was calculated.

### 2.5. Necrosis/Apoptosis Detection

As a complementary assay to evaluate cell viability, we performed necrosis/apoptosis detection in cells exposed to EEP, pinocembrin, or vehicle. RAW 264.5 cells (3.0 × 10^5^/well) were seeded in a 24-well plate in the conditions described above, 12 hours before the experiment. The cells were treated with EEP or pinocembrin at concentrations between 1 and 10 *μ*g/mL under LPS stimuli (100 ng/mL) during 12 hours. The effect of the vehicle was also tested. As a positive control of cell death, we used cells exposed to 5% dimethyl sulfoxide. Treated cells were washed and then resuspended in reaction buffer. Annexin V-FITC and propidium iodide were added following the manufacturer's instructions (Sigma Chemical Company, St. Louis, MO, USA). The cells were incubated for 15 minutes at room temperature and were protected from light. Finally, cells were analyzed using a FACS Canto flow cytometer (BD Biosciences, San José, CA, USA). Data analysis was performed using FlowJo version 9.5.1 software (TreeStar). Cells without fluorescence emission were considered to calculate the relative viability percentage with respect to the control.

### 2.6. Gene Expression

RAW 264.7 cells (4.0 × 10^5^ cells/well) were plated and incubated in the conditions above described during 12 hours to allow cells adherence. Then, the fetal bovine serum content on culture media was reduced to 1% and the cells were stimulated with LPS 100 ng/mL (Sigma, USA) during 12 hours and were coincubated with noncytotoxic concentrations of EEP, pinocembrin, and the corresponding concentration of vehicle. At the end of treatment period, total RNA was obtained using Trizol® reagent following the manufacturer's indications (Invitrogen, Life Technologies, USA) and then quantified using a NanoDrop*™* 1000 spectrophotometer (Thermo Scientific, USA). 1 *μ*g of total RNA was reverse-transcribed using High Capacity RNA-to-cDNA Master Mix (Applied Biosystems. Inc., USA). PCR assays were performed using a 7300 Real-Time PCR System (Applied Biosystems. Inc., USA) in a reaction containing 50 ng of reverse-transcribed RNA, 200 nM concentration of each primer, and 10 *μ*L of 2x Fast SYBR® Green Master Mix (Applied Biosystems. Inc., USA) on a final volume of 20 *μ*L under cycling conditions recommended by the manufacturer. Primers used were MMP-9F 5′-TG CCC ACC GTC CTT TCT TGT T-3′, MMP-9R 5′-TGC TCG GAT GCA TCT GCA ACT-3′, Rpl13aF 5′-TCC TCA AGA CCA ACG GAC TCC T-3′, and Rpl13aR 5′-AAC CTT TGG TCC CCA CTT CCC T-3′ for MMP-9 and Rpl13, respectively. The relative gene expression was analyzed using the qPCR database [28] in which the corresponding Cq was obtained using the Miner algorithm [29]. Rpl13a was used as reference gene.

### 2.7. Gelatinolytic Activity

RAW 264.7 cells (4.0 × 10^5^ cells/well) were plated and incubated in standard conditions during 12 hours. Then, the cells were washed and culture medium was replaced by serum-free media. The cells were activated with LPS (100 ng/mL) and coincubated with EEP, pinocembrin, or vehicle during 24 hours. The supernatant medium was collected and the protein concentration was measured using Modified Lowry Protein Assay Kit (Pierce Biotechnology Inc.). Then, 50 *μ*g of proteins was electrophoresed in a 10% acrylamide gel containing 1 mg/mL gelatin. After separation, gels were washed with 2.5% Triton X-100 and incubated for 18 hours in a reaction buffer (0.05 M Tris–HCl (pH 8), 5 mmol/L CaCl_2_, and 5 mmol/L ZnCl_2_). Finally, gels were stained with Coomassie Brilliant Blue R250. Clear areas indicating gelatin lysis were quantified using the imageJ 1.46r software (National Institute of Health, USA).

### 2.8. Statistical Analysis

Results were analyzed using GraphPad Prism version 5.0a (GraphPad Software, San Diego CA, USA). Data are presented as mean ± SD. Differences between groups involving continuous variables were evaluated by one-way ANOVA with Dunnett's posttest in those comparisons when significant differences were detected. Statistical significance was set at *α* = 0.05.

## 3. Results

### 3.1. Ethanolic Extract of Propolis Content and LC-DAD-MS Analysis

**Figure 1:**
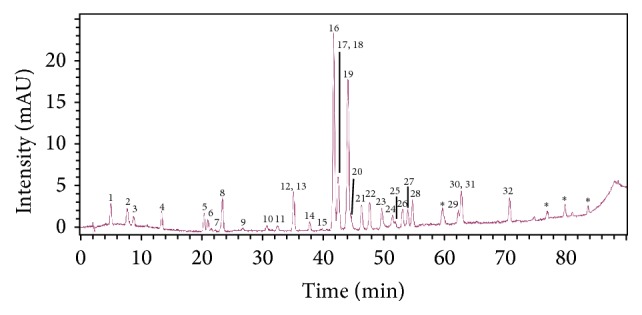
Chromatogram at 290 nm showing the main components found in the ethanolic extract of Chilean propolis. 1: caffeic acid; 2: p-coumaric acid; 3: ferulic/isoferulic acid; 4: 3,4-dimethylcaffeic acid; 5: pinobanksin-5-methyl ether; 6: p-coumaric methyl ester; 7: quercetin; 8: pinobanksin; 9: quercetin-3-methyl ether; 10: pinocembrin-5-methyl ether; 11: apigenin; 12: luteolin-5-methyl ether; 13: cinnamyliden acetic acid; 14: pinobanksin derivative; 15: isorhamnetin; 16: pinocembrin; 17: caffeic acid benzyl ester; 18: caffeic acid isoprenyl ester; 19: pinobanksin-3-*O*-acetate; 20: caffeic acid isoprenyl ester; 21: chrysin; 22: caffeic acid phenethyl ester (CAPE); 23: galangin; 24: chrysin methyl ether; 25: p-coumaric benzyl ester; 26: caffeic acid derivative; 27: pinobanksin-3-O-propionate; 28: caffeic acid cinnamyl ester; 29: pinobanksin-3-O-pentenoate; 30: p-coumaric cinnamyl ester; 31: pinobanksin-3-O-butyrate; 32: pinobanksin-3-O-pentanoate/2-methylbutyrate; *∗*: unknown.

An ethanolic extract of propolis (EEP) was prepared from a propolis sample obtained from southern Chile (Cunco, La Araucanía). The chemical characterization of EEP by liquid chromatography coupled to diode array detection and mass spectrometry (LC-DAD-MS) detected the presence of 36 compounds, successfully identifying 32 of them. The major components found in the extract were pinocembrin and derivatives of caffeic acid and pinobanksin (Figure 1).

### 3.2. EEP Treatment Does Not Affect Cell Viability in RAW 264.7 Cells

**Figure 2:**
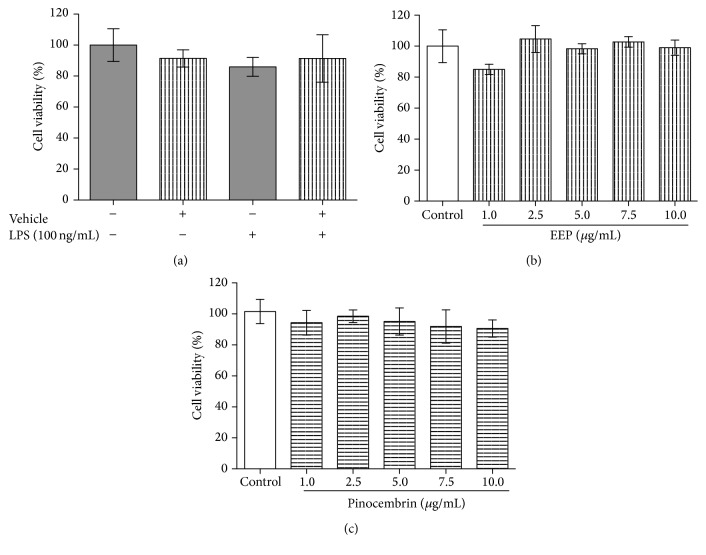
MTT cell viability assay for ethanolic extract of polyphenols, pinocembrin, or vehicle exposed cells. (a) Cell viability of vehicle exposed RAW 264.7 cells in presence or absence of LPS stimuli (100 ng/mL). (b) and (c) Effect of EEP and pinocembrin on cell viability of RAW 264.7 cells under LPS stimulus.

**Figure 3:**
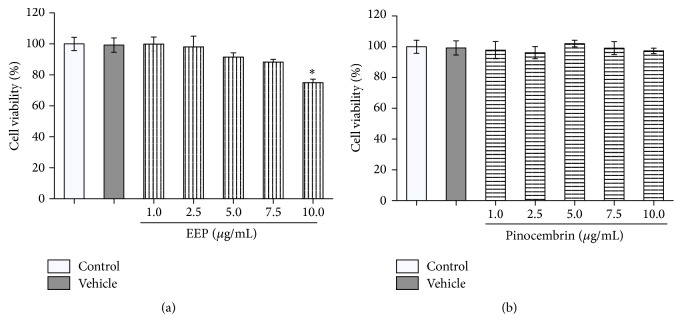
Necrosis/apoptosis detection in activated RAW 264.7 cells exposed to ethanolic extract of propolis, pinocembrin, and vehicle. (a) Effect of EEP (1–10 *μ*g/mL) on cell viability by necrosis/apoptosis detection. (b) Effect of pinocembrin treatment (1–10 *μ*g/mL) on cell viability by necrosis/apoptosis detection in RAW 264.7 cells under LPS stimulus. ^*∗*^ANOVA: *p* = 0.004; Dunnett's multiple comparison test: *p* < 0.05.

RAW 264.5 cells were treated with EEP or pinocembrin at concentrations within 1–10 *μ*g/mL in presence or absence of lipopolysaccharide (LPS, 100 ng/mL) stimuli. The effect of treatment on cell viability was evaluated by 3-(4,5-dimethylthiazol-2-yl)-2,5-diphenyl tetrazolium bromide (MTT) reduction assay.  Figure 2 shows the relative viability of RAW 264.7 cells under experimental conditions described above. The activation of macrophages using LPS did not show variations with respect to control cells. Similarly, vehicle exposition did not alter the cell viability in either presence or absence of LPS stimuli. Regarding treatment exposition of activated RAW 264.7 cells, EEP and pinocembrin did not show significant variations at explored concentrations. Additionally, we assessed cell viability relative to control using a necrosis/apoptosis detection assay, in which no differences were observed up to 7.5 *μ*g/mL of EEP. In pinocembrin and vehicle treated cells, no significant changes were observed (Figure 3).

### 3.3. Inhibition of MMP-9 Expression by EEP Treatment in RAW 264.7 Cells

**Figure 4:**
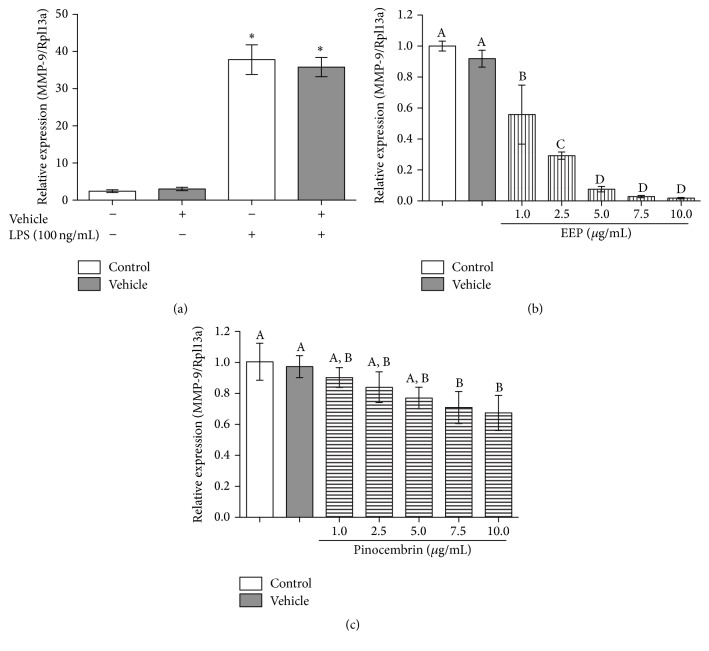
Effect of ethanolic extract of propolis, pinocembrin, and vehicle on relative gene expression of MMP-9. (a) Vehicle induced effect on MMP-9 gene expression in presence or absence of LPS stimulus. (b) Effect of EEP on MMP-9 relative gene expression. (c) Effect of pinocembrin on MMP-9 relative gene expression. Different letters indicate significant differences. ^*∗*^ANOVA: *p* < 0.0001; Tukey's Multiple Comparison Test: *p* < 0.05.

The mRNA expression of MMP-9 in RAW 264.7 cells was evaluated by quantitative real-time PCR (qRT-PCR). The vehicle did not affect MMP-9 gene expression in conditions of LPS-stimulation and unstimulated cells. Moreover, treatment with EEP showed a significant reduction in a dose dependent manner with higher inhibition at the highest concentrations of treatment. Similarly, pinocembrin treated cells showed an inhibitory effect on MMP-9 mRNA expression. However, the effect exerted by pinocembrin treatment was lesser than the effect showed by the EEP treatment (Figure 4).

### 3.4. Inhibition of MMP-9 Activity by EEP Treatment

**Figure 5:**
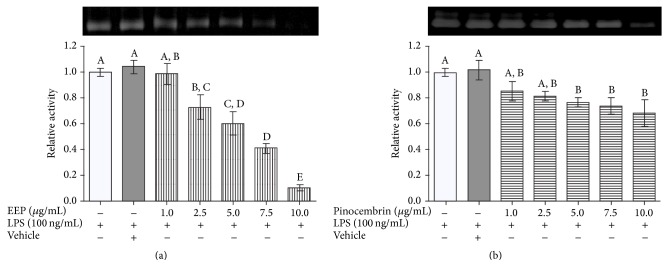
Effect of ethanolic extract of propolis, pinocembrin, and vehicle on gelatinolytic activity of MMP-9. (a) Effect of EEP on MMP-9 relative proteolytic activity. (b) MMP-9 relative proteolytic activity of pinocembrin treated cells. Different letters indicate significant differences.

Finally, we evaluated the effect of EEP and pinocembrin on the gelatinolytic activity of MMP-9 secreted by activated macrophages. In cells treated with EEP, we observed a significant reduction of collagen degradation starting at 2.5 *μ*g/mL, with an increasing effect at higher EEP concentrations. Pinocembrin treated cells also showed a significant reduction of collagen degradation, but to a lesser extent than EEP, affecting the gelatinolytic activity from 7.5 *μ*g/mL of treatment (Figure 5).

## 4. Discussion

Biologic activity of polyphenols from several sources has been widely studied. Among the common sources, propolis offers complex mixtures to evaluate the joint effect of its constituents. The chemical composition of propolis samples is determined by factors as botanical and geographical origin [34]. These factors define a characteristic pattern of compounds, referred to as propolis fingerprinting [35]. South American propolis contains certain predominant compounds as Artepillin C and p-Coumaric acid found in Brazilian propolis [36, 37] and pinocembrin in Chilean propolis [28, 29]. In the present study, we used a propolis sample collected from southern Chile. As aforementioned, pinocembrin was one of the predominant components. However, considering the large amount of caffeic acid and pinobanksin derivatives, probably these two compounds could also influence its biological activities and should be studied separately.

Evidence of biological activity of Chilean propolis has been shown in previous works by our group [38–41]. The present study demonstrates an inhibitory effect of EEP and pinocembrin on both gene expression and gelatinolytic activity of MMP-9 using a cellular model of activated macrophages (LPS 100 ng/mL). LPS stimuli result in the induction of numerous inflammatory mediators as cytokines and chemokines including TNF-*α*, IL-1, IL-6, and MCP-1. This effect is associated with the activity of inflammation-related transcription factors as NF-kappa B [42], also involved in MMP-9 expression [43]. In atherosclerosis development, matrix metalloproteinases are mainly secreted by macrophages. These enzymes are involved in vascular remodeling allowing the adaptation of affected vessel to the vascular injury in order to maintain the lumen diameter, mechanism modulated by wall components and extracellular matrix, especially by its degradation. However, MMP-9 proteolytic activity has been associated with the progression of atherosclerotic plaques to a vulnerable state and consequently to the development of ischemic events [10, 44–47]. Matrix metalloproteinases are also involved in other disease-associated processes such as cell invasion and metastasis in cancer [48]. In this context, using an* in vitro* model of hepatocellular carcinoma, treatment with polyphenols from propolis in concentrations similar to those used in the present work did inhibit the activity of MMP-9, similar to the effect associated with caffeic acid phenethyl ester treatment obtained from the propolis sample [49]. This effect on MMP-9 has also been demonstrated by other isolated compounds as kaempferol, apigenin, resveratrol, and quercetin [50, 51]. Pinocembrin exhibits antibacterial, anti-inflammatory, anticancer, and neuroprotective activities [30], and the anti-inflammatory effect exerted by pinocembrin has been associated with suppression of I*κ*B*α*, JNK, and p38MAPK activation [30, 52], signaling pathways involved in MMP-9 induction in LPS-stimulated macrophages [53, 54]. Our study compared the effect of pinocembrin and EEP, obtaining an inhibitory effect on both gene expression and proteolytic activity. However, the modulation demonstrated using this particular flavonoid as treatment was lesser than that exhibited by EEP, which has, among its components, detectable amounts of all compounds listed above except for resveratrol. So, the observed effect can be a product of the joint activity of identified compounds. In conclusion, polyphenolic components of Chilean propolis show a significant inhibition of MMP-9 gene expression and activity, suggesting a potential role in the control of extracellular matrix degradation in atherosclerotic plaques and subsequently on plaque stability.

## 5. Conclusion

In summary, our results indicate that components of Chilean propolis showed a significant inhibition of MMP-9 gene expression and activity, suggesting a potential role in the control of extracellular matrix degradation in atherosclerotic plaques and subsequently on plaque stability.
